# Weak Static and Extremely Low Frequency Magnetic Fields Affect *In Vitro* Pollen Germination

**DOI:** 10.1100/tsw.2011.83

**Published:** 2011-04-19

**Authors:** Lucietta Betti, Grazia Trebbi, Fabio Fregola, Michela Zurla, Pietro Mesirca, Maurizio Brizzi, Francesco Borghini

**Affiliations:** ^1^ Department of Agro-Environmental Science and Technology, Faculty of Agriculture, Bologna University, Italy; ^2^ Department of Evolutionary Experimental Biology, Faculty of Science, Bologna University, Italy; ^3^ Department of Physics, Faculty of Science, Bologna University, Italy; ^4^ Department of Statistical Sciences, Faculty of Statistical Sciences, Bologna University, Italy; ^5^ Department of Medical Therapy, Faculty of Medicine, Chieti University, Italy

**Keywords:** static weak magnetic fields, extremely low frequencies, in vitro pollen germination, water-mediated effects

## Abstract

This study concerns the effects of a weak static magnetic field (MF) at 10 μT oriented downward, combined with a 16-Hz sinusoidal MF (10 μT), on *in vitro* pollen germination of kiwifruit (*Actinidia deliciosa*). Extremely low frequency magnetic field (ELF-MF) exposure was carried out by a signal generator unit connected to a copper wire solenoid, inside which samples where placed. Two different kinds of treatment were performed: direct and indirect. In the direct treatment, pollen samples were directly exposed during rehydration, germination, or both. In the indirect treatment, the pollen growth medium was prepared with water aliquots (at standard temperature of 20°C and pH = 6.74) that were exposed before use for 8 or 24 h. The main purpose of our research was to identify a biological marker (*in vitro* pollen germination in a stressing growth medium without Ca^2+^) susceptible to the effects of direct or indirect ELF-MF exposure. The working variable was the pollen germination rate, as detected blind after 3 h 30 min by an Axioplan microscope. A directionally consistent recovery of germination percentage was observed both for direct exposure (during germination and both rehydration and germination phases) and water-mediated exposure (with water exposed for 24 h and immediately used). Our results suggest that the ELF-MF treatment might partially remove the inhibitory effect caused by the lack of Ca^2+^ in the culture medium, inducing a release of internal Ca^2+^ stored in the secretory vesicles of pollen plasma membrane. Although preliminary, findings seem to indicate the *in vitro* pollen performance as adequate to study the effects of ELF-MFs on living matter.

### INTRODUCTION

The biological effects of weak extremely low frequency magnetic fields (ELF-MFs) on humans, animals, and micro-organisms have been intensively studied. In the last half-century, both *in vivo* and *in vitro* effects on plants have also been investigated, as reviewed by Galland and Pazur[1]. More recently, biochemical changes induced by ELF-MFs on different plant species[2,3,4,5,6] and on tobacco resistant to tobacco mosaic virus[7] have been reported. Magnetoreception in plants has been related to different mechanisms, such as ferrimagnetism[8] and electron spin–controlled chemical reactions by radical pairs[9,10]. Nevertheless, considering that MF effects could be observed in organisms without proven ferromagnetic particles and at field strengths well below those required for the radical pair reaction, other mechanisms have been considered[11]. In particular, "windows" of optimal effectiveness have been seen for certain combinations of field strengths and frequencies; this nonlinear dose-response effect was related to ion cyclotron resonance (ICR) of small ions[12,13,14,15,16,17].

In the interaction between the bio-object and electromagnetic fields (EMFs), water seems to play a key role owing to its particular properties; changes in its structure induced by exposure to EMFs were directly detected by instruments in many studies, leading to the hypothesis of water as the first receptor and mediator of EMF effects on biological systems[7,18,19,20,21,22,23,24,25].

Nevertheless, there is a growing body of research using low-energy EMFs, but the results are usually small and reproducibility is a problem. A suitable model is needed that is relatively easy for other scientific teams to master. The present study was planned to provide experimental evidence for the bio-effects of weak ELF-MFs, using *in vitro* pollen germination as a "sensor" model, lacking the placebo effect, and available in large populations to ensure accurate statistical analysis. To the best of our knowledge, only a few investigations have been performed on this topic[26,27,28].

Pollen germination and tube growth, based on a complex, sensitive, and well-regulated network of structures (Fig. 1) and functions[29,30,31], represents an excellent model for the study of growth and morphogenesis at the cellular level. In fact, pollen, which plays a key role in reproductive biology, is a highly reduced and dehydrated structure comprising only two or three cells when it is released at anthesis; after its landing on stygma, it rehydrates and elongates a pollen tube within the female tissues. Therefore, throughout the *in vivo* progamic phase or *in vitro* pregermination, water inside the pollen grain plays a very important role, at the basis of efficient germination and tube growth. Moreover, during these events, the role of $K^+$, $Cl^-$, and especially $Ca^{2+}$ ions must also be stressed[32,33]. In fact, in growing pollen tubes, a $Ca^{2+}$ gradient and a positive correlation between changes in the concentration of cytosolic free $Ca^{2+}$, and changes in the rate and direction of pollen tube growth, have been reported[32,34]. The main processes under $Ca^{2+}$ control during pollen germination and tube growth are microtubule polymerization, permeability properties of the tube membrane, modulation of mitochondrial metabolism, and enzyme secretion.

Although there is no direct information on the target components of weak electromagnetic exposure in the "pollen system", calcium ions could constitute one of the possible targets; in fact, $Ca^{2+}$ transport and binding is the parameter most consistently suggested to be influenced by ELF fields[17,35,36]. In particular, it was reported that a static MF can enhance the transport of $Ca^{2+}$ across the cell membrane and, as a consequence, alter pollen germination[27]. Furthermore, characteristic anomalies related to changes in the ionic charges within the pollen tube cytoplasm were observed in pollen tube growth in the presence of an alternating MF at 50 Hz[28]. Finally, it was reported that pollen growth can be influenced by application of weak electric fields[37].

Since pollen culture is relatively easy to manage, pollen tubes have become a useful *in vitro* system as a paradigmatic example of tip growth[29,38]. They may thus be used to highlight mechanisms similar in other cells, including fungal hyphae, root hairs, and other plant cells, as well as certain specialized animal cells. Moreover, owing to the oscillatory behavior of pollen tube growth, this model seems to be well suited to the study of various aspects of nonlinear dynamics in biological systems[32,39].

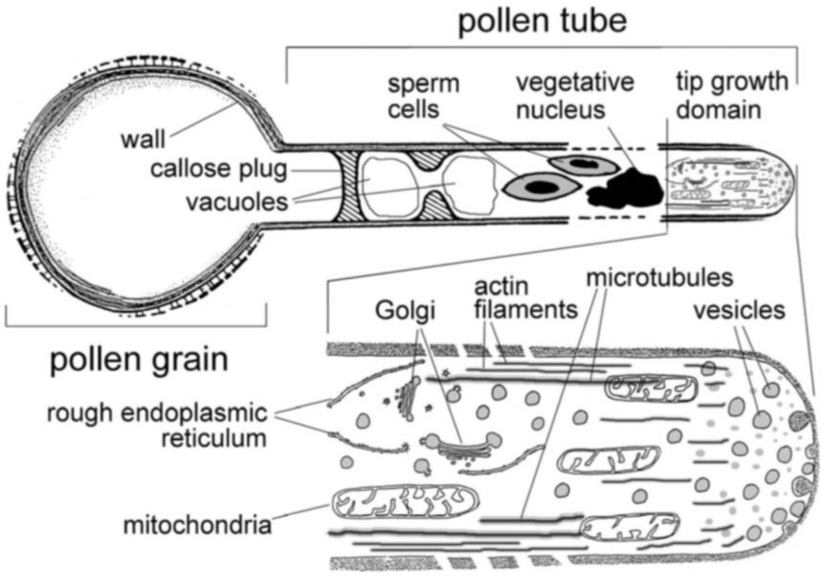

**FIGURE 1**. Schematic drawing of germinating pollen grain.

The most appropriate protocol for running experiments was evaluated in the present study, with the aim of better clarifying the correlations between the pollen germination process and an electromagnetic stimulus. Pollen of kiwifruit (*Actinidia deliciosa*) was either directly or indirectly exposed to a combination of a weak static MF (magnitude 10 µT) and a 16-Hz sinusoidal MF (10 µT). Since several studies[18,19,20] reported that both static MF and ELF-MF had biological effects on the process of seed hydration, solubility, water binding in seed, and germination, we decided to test the effects of a combination of weak static MF and ELF-MF. The intensity of static MF was chosen as comparable to the range limits of the geomagnetic field in an everyday environment[1,40]. Besides the intensity and the kind of signal used, the effects of ELF-MF critically depend on the frequencies employed; it has been reported that MF exposures at very low intensity (20 µT), modulated at ELF in series from 50, 16.66, 13, 10, 8.33 to 4 Hz, could alter relative power within the corresponding EEG bands[41,42]. Moreover, in previous works, the authors reported an efflux of calcium ions in response to a weak very high frequency (VHF) field modulated at 16 Hz either in isolated brain of neonatal chick[43] or in intact myocardium of frog[44]. Finally, exposure to 147-MHz carrier waves at 0.05 W/kg, amplitude modulated (AM) at 16 Hz, induced enolase activity enhancement in *Escherichia coli* cultures, whereas AM at 60 Hz showed enolase activity reduction[45]. Although the underlying basis of these effects has not yet been established, the above cited results seem to show a differential sensitivity of the bio-object to different frequencies.

Since several experiments showing an effect due to an ELF-MF exposure included biotic or abiotic stresses[7,46], in the present experiment, we used a stressing growth medium without $Ca^{2+}$ in order to partially inhibit the pollen tube emergence and verify if ELF-MF exposure at 16 Hz could allow recovery of a standard germination value. Moreover, the biological effects on pollen germination induced by ELF-MF–exposed water used to prepare the growth medium were evaluated. The main purpose of our research was to identify a biological marker (pollen germination in a stressing growth medium without $Ca^{2+}$) susceptible to the effects of direct or indirect exposure to weak static and ELF-MFs, regardless of the hypothetical physicochemical mechanisms implied in it; a detailed list of such kind of mechanisms has been widely reported by Chaplin[47]. Hence, we provide preliminary evidence that pollen growth processes are significantly affected either directly through ELF-MF treatment of the grains or indirectly by ELF-MF exposure of the water of the culture medium.

## MATERIALS AND METHODS

### MF Generation

ELF-MF exposure was carried out by a device of local design already used in our previous work[7]. The signal generator unit is connected to a solenoid consisting of a copper wire, 1 mm in diameter, wound around a cylinder made of nonconducting material (71 turns, 300 mm diameter, 345 mm high). The signal generator produces (1) a static MF ($B_0$) of low intensity (0–60 μT) and (2) a sinusoidal MF ($B_c$), at perfectly stabilized frequencies ($v$), from 3 to 83 Hz, with intensities ranging between 0 and 36 μT. The current generation is made from a simple frequency-modulated power supply unit overlapping a direct current (DC) base made from a dedicated power supply. In this way, we are able to have both static and alternate current (AC) flowing in the solenoid simultaneously, generating static and modulated fields. Both intensity and frequency can be set at fixed values. Samples were placed on a nonconducting holder in the center of the coil, where the MF is highly uniform; current distribution inside the sample holder was simulated using Comsol Multiphysics software. From the simulation, the current induced resulted perpendicular to the direction of the MF and it can be represented with concentric lines in the plane of the Petri dishes. In each trial, the temperature was monitored by a couple of thermoresistors, alumel-chromel, connected to a thermometer (model HD8704, DeltaOhm, Padova, Italy). The results showed no significant differences in temperature inside the culture medium between exposed (inside the solenoid) and nonexposed samples (outside the solenoid).

The background MF due to the AC component and the local static geomagnetic field in the lab was measured. The value of background MF was in the region of 0.2 μT as measured by the highly sensitive EMDEX II probe (Enertech Consultants, Campbell, CA, USA). The local static geomagnetic field, measured by a gaussmeter (model 7010, F.W. Bell, Orlando, FL, USA), was evaluated inside the solenoid and 2 m away from it (where the control samples had to be placed). By measuring x, y, and z components, a value of 39 ± 1 μT was detected both inside and outside the solenoid; the field was essentially directed along the vertical z axis and oriented downwards, the value of the z component being -35 μT inside and -40 μT outside the solenoid. In this study, a static MF, at 25 ± 2 μT (in z direction oriented upwards), combined with a sinusoidal MF at 16 Hz at 10 ± 0.2 μT, was used. Taking into account that inside the solenoid the static MF along the z axis is the combination of the local geomagnetic field (-35 μT) plus the static MF produced by the signal generator (25 μT), the total static MF was 10 ± 1 μT oriented downward.

### Plant Material

Freshly opened flowers from the male genotype of *A. deliciosa* (A. Chev.) C.F. Liang and A.R. Ferguson var. *deliciosa* (cv. Tomuri) were collected in experimental plots near Faenza (Italy) in two different years (2007 and 2008). The anthers obtained from flowers were allowed to dehisce under controlled conditions for about 20 h, exposed to a temperature of 28°C and 40% relative humidity. Pollen, collected through a sieve, was then recovered and stored in glass containers at –20°C under NaOH pellets[48].

### *In Vitro* Pollen Culture

Before germination, pollen was subjected to a rehydration phase in a glass Petri dish at 100% relative humidity for 30 min at 30°C. Germination was performed in glass Petri dishes (5 cm diameter), suspending pollen in a standard liquid growth medium containing 0.29 M of sucrose, 0.4 mM of boric acid, and 1 mM of calcium nitrate[49,50]. The pollen suspension was at 1 mg/ml, which is the optimal ratio between the pollen weight and the growth medium volume in order to obtain the top values of *in*

*vitro* germination[48]. Pollen suspension was equally subdivided in Petri dishes (3 ml each) and germination was performed for 3 h at 30°C in the dark as previously described[48].

Preliminary studies were carried out to assess the effect on pollen germination of the suspension volume, the duration of the germination phase, and any influence of the Petri dish component (plastic or glass).

### Image Detection

After 3 h of *in vitro* germination, a fixed number of digital images of nonoverlapping fields was taken from every Petri dish, moving along three separate radii (four equally distanced frames for each radius). This pattern was defined taking into account that the spatial distribution of pollen grains within the Petri dishes was not perfectly homogeneous, as the central area showed a higher concentration than peripheral zones. A video camera SSC-M370CE (Sony, Tokyo, Japan) connected to an Axioplan microscope (Zeiss, Ober Kochen, Germany), managed by Imaging System KS100 (Kontron, Eching, Germany) software, was used. In a set of preliminary tests, we also took into account the optimal microscope lens for image detection and the subsequent evaluation of germination rate, with the aim of obtaining the highest number of pollen grains per optical field and a sharpness level suitable to allow reliable pollen grain counting. This goal was obtained with a 4× lens, which made it possible to collect an average number of 150 pollen grains in each frame. For every digital image, the number of both total and germinated pollen grains was detected by the experimenter using specific software, after assigning black dots to ungerminated pollen grains and white dots to germinated grains; the program also provided the germination rate per frame (Fig.2). The data were then pooled with homologous values of other frames. Only pollen grains producing a tube equal to or longer than the grain diameter (30–35 μm) were considered as germinated.

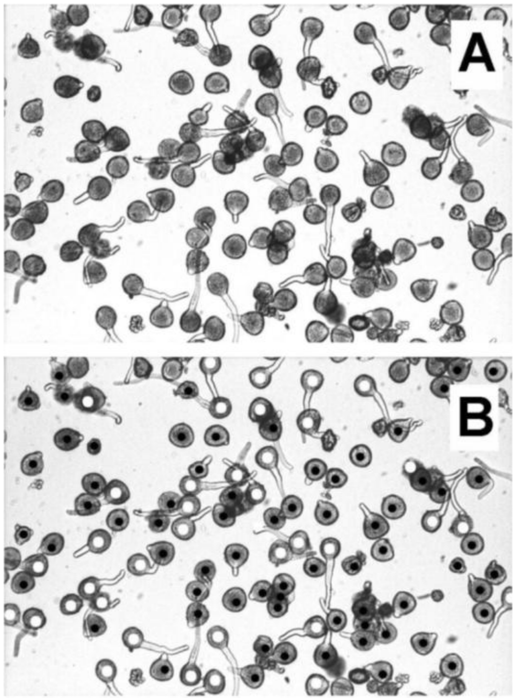

**FIGURE 2**. Example of image used for data recording, before (A) and after (B) processing. Ungerminated pollen grains (black dots) and germinated grains (white dots) are shown in B.

In order to evaluate experimenter bias in pollen grain counting procedures, a sample of ten frames was evaluated by two different experimenters and their counting results were statistically compared. For a clearer evaluation of experimenter bias, a further blind trial was carried out, where the same images were read twice by the same experimenter after a lag of a few minutes.

### Negative Control Experiments

We checked negative controls by separately testing pollen collected in 2007 and 2008, to verify the reproducibility of germination rate in standard conditions. Two independent experiments (three Petri dishes each) were carried out for each pollen collection.

### Choice and Evaluation of Stress Conditions

To evaluate a possible effect of ELF-MF exposure on germination of pollen grains, we tried to define the most suitable stress conditions for inducing a repeatable reduction of germination rate. After checking different physical and chemical stresses, we observed that the most stable and regular inhibition was obtained by reducing the level of calcium in the growth medium. We therefore tested both pollen 2007 and 2008 using two different levels of calcium nitrate (0.5 and 0 mM) with respect to the standard concentration (1 mM), utilizing three Petri dishes for each level.

To verify the reproducibility of the germination rate in the absence of $Ca^{2+}$, two negative control experiments (three Petri dishes each) were carried out for each pollen collection (2007 and 2008).

### Experimental Design: ELF-MF Exposure

Two different kinds of treatment were performed: direct and indirect. In direct treatments (Fig. 3A), pollen samples were directly exposed to the combination of static (10 ± 1 µT oriented downward) and sinusoidal MFs (16 Hz, 10 µT). This exposure was performed either during rehydration (30 min), germination (3 h), or both (3 h 30 min). In indirect treatments (Fig. 3B), the pollen growth medium was prepared with water aliquots (at standard temperature of 20°C and pH = 6.74) that were exposed before use for 8 or 24 h to the static MF at 10 µT oriented downward, plus the 16-Hz sinusoidal MF at 10 µT. In all cases, aliquots of nonexposed water were used for control medium. Conditioned water was utilized 0, 1, or 8 h after the end of ELF-MF exposure.

In each trial, two experimental groups (control and treated pollen) of four Petri dishes each were compared; in all cases, pollen growth medium was prepared without calcium. Each experiment was independently replicated three times on different days. In direct treatments, Petri dishes of control groups were always kept outside the solenoid (2 m from the device).

Experiments were blinded with coded samples; a person not involved in carrying out the experiments coded the samples with a random letter code. The code was kept secret until all measurements and basic data processing were completed.

### Statistics

The experimental variable was the percentage frequency of germinated pollen grains (number of germinated grains with respect to the total) for each frame; data of germination correspond to at least 5000 pollen grains for each treatment group (~150 pollen grains/image, 12 images/dish, three dishes/treatment group). Treatment effects were evaluated by means of a test of comparison between two frequencies with normal approximation, suitable for binomial analysis of a large dataset[51]. The null

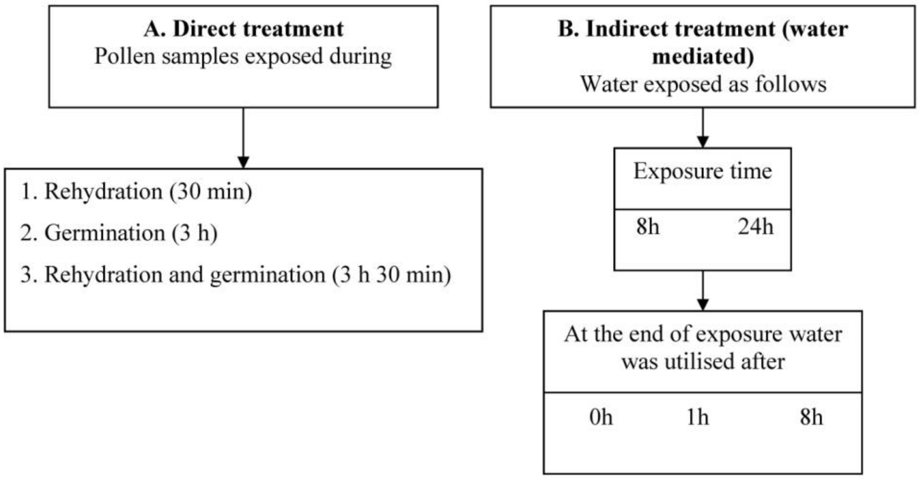

**FIGURE 3.** Different steps of direct (A) and indirect (B) treatments.

hypothesis $H_0$ is that frequency of germinated pollen grains is the same for exposed and control. If we denote the observed frequencies of germinated pollen grains with $f_C$ (control) and $f_E$ (exposed), and the corresponding sample sizes with $n_C$ and $n_E$, we can compute the global frequency:

$$f^* = \frac{f_C n_C + f_E n_E}{n_C + n_E}.$$

The test statistics to be applied for judging $H_0$ are the following:

$$z = \frac{f_E - f_C}{\sqrt{f^*(1 - f^*)\frac{n_C + n_E}{n_C \cdot n_E}}}$$

Since the sample size is, in every experiment, large enough to allow a normal approximation, we can use standard normal tables to check whether the value of z, representing the difference of frequencies, is significant or not. In the first case, we will reject $H_0$. In each comparison, we indicate here the resulting value of z, specifying when the results are significant.

The comparison between frequencies is graphically represented for easier reading. For each frequency, we also indicate the corresponding standard error; indeed, given a frequency $f$ and a sample size $n$, the standard error $s(f)$ is equal to

$$s(f) = \sqrt{\frac{f(1-f)}{n}}.$$

### RESULTS

#### Setup of *In Vitro* Pollen Culture

Preliminary trials were carried out, testing pollen collected in 2007 and 2008, in order to fix the experimental parameters yielding the highest possible pollen germination rate. First of all, we evaluated the suspension volume effect in the Petri dishes, taking into account the possibility of a sort of choking effect of pollen grains located in the deepest layers. Having this aim, we compared two different volumes (5 and 3 ml) of pollen suspension (three Petri dishes per volume); the results showed a germination rate significantly higher when using the smaller volume (82 against 54%, z = +22.001), which was finally adopted in our experimental protocol.

Another parameter to be determined was the optimal duration of the germination phase for kiwi pollen. Consequently, we studied the pollen germination curve as a function of time (Fig. 4). The data (for both 2007 and 2008 pollen) showed a germination peak after 3 h, which was then chosen as the most convenient time for image detection. In fact, after such an "optimal" time, a decrease in the germination rate was detected, due to some degenerative processes, such as pollen tube deformity and/or bursting.

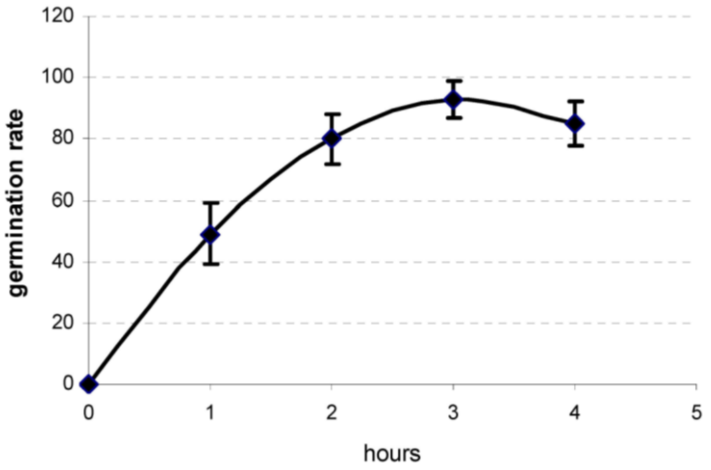

**FIGURE 4**. Germination curve of kiwi pollen according to time.

Finally, a possible influence of the Petri dish component (plastic or glass) was checked, comparing three Petri dishes for each kind of material. No significant differences were detected between plastic and glass dishes (z = +0.187), so we decided to use glass Petri dishes, as done by most laboratories working in the same field of research.

### Image Detection

In another series of preliminary trials, we searched for the "best" total number of digital images per Petri dish, in order to obtain a good reproducibility of results associated with the shortest period of experimental work. We began with a total number of 60 digital images per Petri dish, taken moving along six separate radii (10 equally distanced frames for each radius). After randomly dividing these images into two groups of 30 images each, no significant differences were found between the groups analyzed (z = +0.538). The result of the statistical test indicates that it is not worth shooting more than 30 digital images per dish. The total number of images was thus reduced to 24 (eight equally distanced frames for each of three radii) and even here the difference between two randomly distributed 12-image groups was not significant (z = +1.035). The experimental protocol finally adopted, therefore, consisted of 12 digital images per Petri dish, taken moving along three separate radii.

Experimenter bias was checked by a statistical analysis (z test) of pollen germination rates in standard conditions. We compared the counting results of ten frames and we did not detect any significant difference, since the largest z value observed was 0.535, very far from significance. Moreover, when comparing the results of the two analyses performed blind on the same frames, by the self-same experimenter, after a few minutes, no significant differences were detected (z = +0.219). The influence of the experimenter can thus be considered negligible.

### Negative Control Experiments

Statistical analysis was first performed to evaluate any differences between germination rates of control groups in standard conditions (Fig. 5); pollen germination was about 68 and 77% for the pollen collections 2007 and 2008, respectively. Comparison of frequencies did not reveal any significant difference (2007: z = +0.976; 2008: z = +0.561). We thus concluded that the experimental system was reliable and did not produce false-positive results for identical treatment parameters.

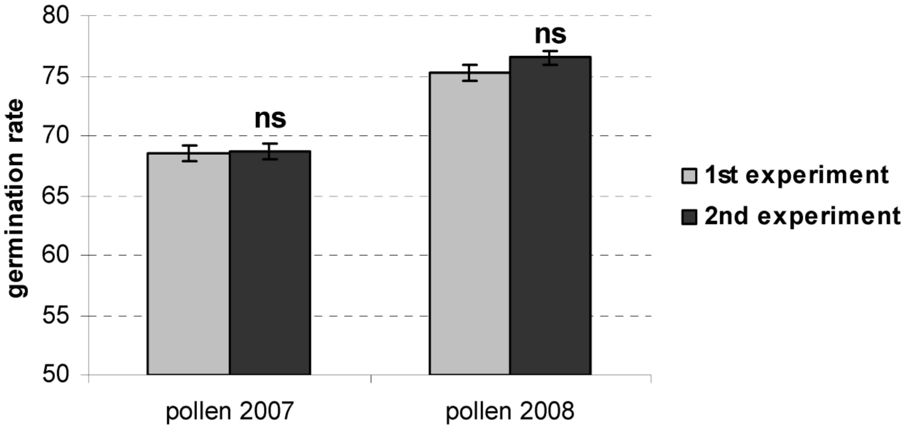

**FIGURE 5**. Negative controls in standard growth medium (1 mM $Ca^{2+}$) for 2007 and 2008 pollen collections. n.s. = no significant difference (z test).

### Stress Evaluation

Once the most suitable kind of stress to apply had been defined, the effect on pollen germination of a reduction in $Ca^{2+}$ molarity (0.5, 0 mM) in the growth medium was tested in comparison with $Ca^{2+}$ standard molarity (1 mM). Results are shown in Fig. 6; following the reduction of $Ca^{2+}$ molarity, a highly significant inhibition was revealed. In particular, pollen germination was inhibited approximately by 8% for both pollen collections at 0.5 mM of $Ca^{2+}$, while at 0 mM of $Ca^{2+}$, the inhibition reaches 12% for 2007 and 16% for 2008. Thereafter, it was decided not to add $Ca^{2+}$ at all to the growth medium, in order to evaluate the possible recovery effect following the ELF-MF exposure.

The statistical evaluation of the reproducibility of pollen germination rates at 0 mM of $Ca^{2+}$ did not show any significant difference (2007: z = +1.435; 2008: z = +0.281). We can thus be confident that our experimental system is stable even in the absence of calcium in the growth medium.

### Effects of ELF-MF Exposure

In the experiments described here, the pollen collected in 2008 was chosen, owing to its higher germination rate shown in negative control experiments (Fig. 5).

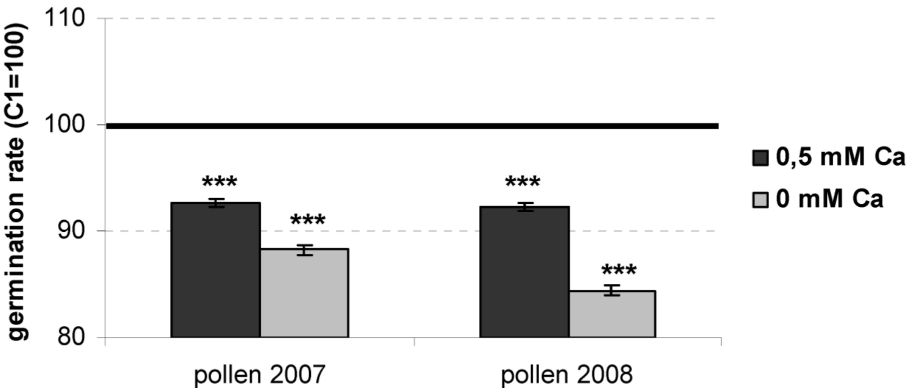

**FIGURE 6**. Effect on pollen germination of different $Ca^{2+}$ molarities (0.5, 0 mM) in the growth medium with respect to pollen germinated at standard conditions (1 mM $Ca^{2+}$, C1 = 100). ***$p \leq 0.001$ (z test).

### *Direct Treatment*

The effects on germination after the direct exposure of pollen to a combination of static (10 ± 1 µT oriented downward) and sinusoidal MFs (16 Hz, 10 µT) at different culture stages (Fig. 3A) are shown in Fig. 7. When pollen was exposed only during rehydration (Fig. 7A), a highly significant inhibition of germination rate was detected in each of the three trials carried out (z = -6.568; z = -6.838; z = -2.690). On the contrary, a significant improvement in germination rate (z = +2.906; z = +5.811; z = +4.751) was shown in every trial when pollen exposure was performed during the 3 h of germination (Fig. 7B). Finally, an even more evident stimulating effect on pollen performance (changes from stressed control values between 8 and 28%) was detected after exposure applied during both rehydration and germination stages (Fig. 7C). In fact, the germination improvement was always highly significant (z = +17.653; z = +4.669; z = +9.889), abolishing the stress of calcium absence.

### *Indirect Treatment*

Results on pollen germination in growth medium, prepared using water previously exposed to the combination of static (10 ± 1 µT oriented downward) and sinusoidal MFs (16 Hz, 10 µT) (Fig. 3B) are reported in Figs. 8 and 9. Water exposed for 8 or 24 h was used either immediately or 8 h after the end of the exposure. Moreover, water exposed for 24 h was tested also 1 h after the end of the exposure.

When germination growth medium was prepared with water exposed for 8 h, no evident trend towards stimulating or inhibiting effects on pollen germination was detected (Fig. 8A,B). In particular, when growth medium was immediately prepared at the end of water exposure (Fig. 8A), germination rate significantly increased 2 times over 3 (z = -4.370; z = +7.496; z = +7.650), while after an 8 h wait, we detected very heterogeneous effects (z = -7.295; z = +6.547; z = +0.368). When germination growth medium was prepared with water exposed for 24 h and immediately used after the end of exposure (Fig. 9A), a consistent and evident stimulating trend was obtained (changes from stressed control values between 4 and 13%; z = +6.422; z = +12.095; z = +4.304); this reproducibility disappears (z = -5.57; z = +12.26; z = -4.68) when water was used after an 8 h wait (Fig.9B).

Since the results obtained using a growth medium prepared 8 h after the end of water exposure did not show any directional trend, irrespective of time-water exposure (Fig. 8B and 9B), we decided to test a shorter wait time. We therefore prepared a growth medium with water exposed for 24 h (which gave the most reproducible results when used immediately), waiting for 1 h before preparing the growth medium. The

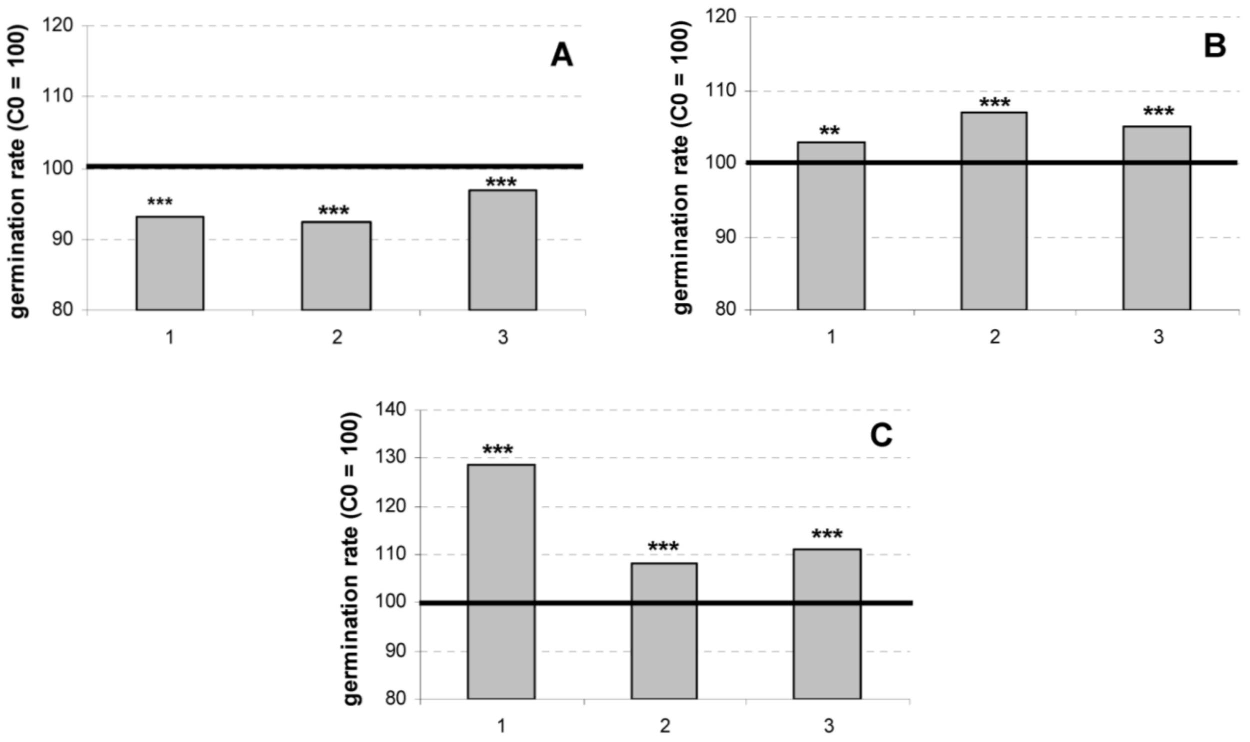

**FIGURE 7**. Effect of combination of static (10 µT) and sinusoidal MFs (16 Hz, 10 µT) on pollen germination in stress conditions (0 mM $Ca^{2+}$) with respect to stressed untreated control (C0 = 100). Direct pollen treatments: MF exposure during rehydration (A), germination (B), both rehydration and germination (C). Experiments 1, 2, and 3 mean repetitions of the same trial. $**p \leq 0.01$; $***p \leq 0.001$ (z test). Standard error is ±0.01 (too small for graphical representation).

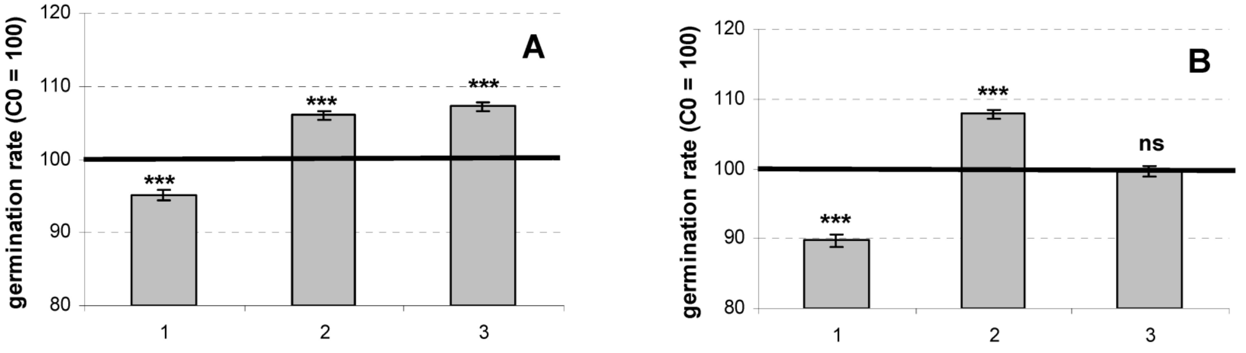

**FIGURE 8**. Effect of combination of static (10 µT) and sinusoidal MFs (16 Hz, 10 µT) on pollen germination in stress conditions (0 mM $Ca^{2+}$) with respect to stressed untreated control (C0 = 100). Indirect pollen treatments with water exposed for 8 h: growth medium prepared immediately (A) and after an 8 h wait (B), from the end of water exposure. Experiments 1, 2, and 3 mean repetitions of the same trial. $***p \leq 0.001$; ns = no significant difference (z test).

results reported in Fig. 9C show a loss of reproducibility also in this case; germination rate significantly decreased 2 times over 3 (z = -4.260; z = -9.011; z = -0.890).

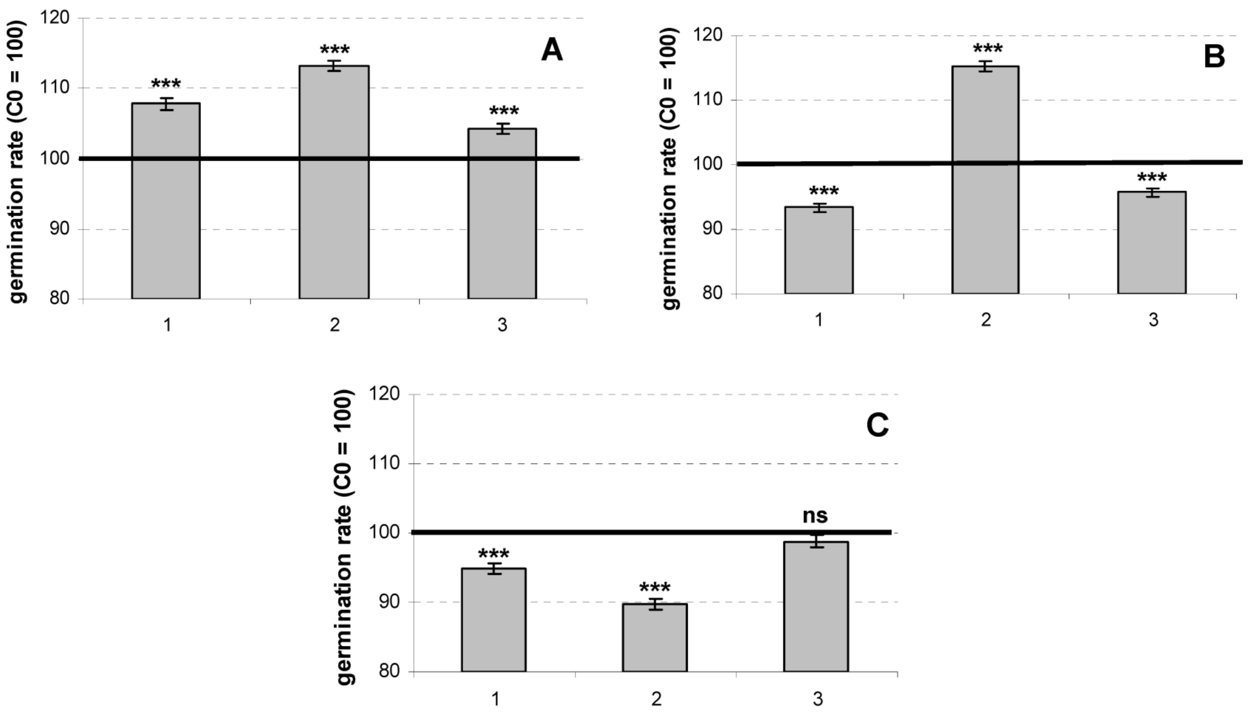

**FIGURE 9**. Effect of combination of static (10 μT) and sinusoidal MFs (16 Hz, 10 μT) on pollen germination in stress conditions (0 mM $Ca^{2+}$) with respect to stressed untreated control (C0 = 100). Indirect pollen treatments with water exposed for 24 h: growth medium prepared immediately (A), after an 8 h wait (B), or 1 h wait (C) from the end of water exposure. Experiments 1, 2, and 3 mean repetitions of the same trial. ***$p \leq 0.001$; ns = no significant difference (z test).

### DISCUSSION AND CONCLUSIONS

*In vitro* pollen performance may represent the spatial and temporal organization of many other developing cells, at least those that expand or differentiate through tip growth, and it can thus become a standard, applicable to other systems. Considering the importance of applying simple approaches to deal with complex and partially unknown research fields, we again emphasize the efficacy of *in vitro* pollen performance as an experimental model appropriate to such a theoretical challenge. In fact, being aware of the controversies about effects of weak intensity ELF-MFs on living matter, one of our purposes was to assess such interactions in organisms lacking the placebo effect and available in large populations. Plant-based *in vitro* models, pollen included, fulfilled these requirements in general.

Before discussing the results we obtained, it is worth pointing out that an essential feature of the pollen model presented here is the possibility of collecting a very large base of data in a relatively short time; this aspect has a primary importance from a statistical point of view, since it makes it possible to apply all the statistical methods requiring a large sample of data. The availability of a large database is surely a great advantage for the reliability of statistical analysis, but it sometimes involves the need for careful reading of the test results. Actually, the dramatically significant values recorded in our experiments are partially due to the extra-large size of our samples, for which the test procedures become strongly sensitive even to small differences. It is therefore very important to take the directional consistency of our results into particular account, without focusing excessive attention on the significance level. Moreover, we characterized the model system in regard to possible artifacts in scoring pollen germination. This is very important for demonstrating that this model might be suitable for continued research investigating the actions of ELF-MFs.

The present paper shows that either direct or water-mediated exposure of pollen grains to weak static MFs (magnitude 10 µT) plus 16-Hz sinusoidal MFs (10 µT) can have significant effects on pollen tube emergence. In both kinds of our experiments, pollen grains were grown in a stress condition, the growth medium lacking in $Ca^{2+}$ ions, which are essential components for standard germination and tube growth[32,39,52]. It is known that growing pollen tubes possess an oscillating "tip focused" gradient (mainly due to cytosolic calcium and, to a lesser extent, to $H^+$, $K^+$ and $Cl^-$ ions), which shows maximum value at the extreme apex of the growing tube[39,52]. This gradient is largely derived from influx of extracellular $Ca^{2+}$ oscillating in magnitude over time and, to a lesser extent, from a release of $Ca^{2+}$ from internal stores[32]. In fact, there are elements of the endoplasmic reticulum in the apical domain that could release $Ca^{2+}$[53], but a more likely candidate would be secretory vesicles closely appressed to the inner surface of the plasma membrane[32]. Our results suggest that the ELF-MF treatment might partially remove the inhibitory effect caused by the lack of $Ca^{2+}$ in the culture medium; a directionally consistent recovery of germination percentage was observed both for direct exposure (during germination and both rehydration and germination phases) and water-mediated exposure (with water exposed for 24 h and immediately used). To explain our findings on pollen performance, we can hypothesize that direct or indirect treatments of pollen grains could have contributed to the release of internal $Ca^{2+}$ stored in the secretory vesicles of the plasma membrane; in fact, it has been proposed that weak ELF-MFs at 16-Hz frequency (resonant frequency for $K^+$ in the Earth's MF) specifically increase the ability of $K^+$ ions to bombard cell membranes and replace bound $Ca^{2+}$[54]. Since in our experimental conditions we observed a germination increase at 10 µT static MF oriented downward, it could be hypothesized that the 16-Hz frequency may induce biological effects also in a range below the limits of the Earth's MF. However, it may be observed that germination was significantly impaired when pollen grains were exposed to direct MF treatment during rehydration. This inhibitory effect could be explained considering that rehydration, which induces pollen imbibition changes from about 10 to 70%, is a very sensitive phase in pollen performance. In fact, during the first minutes of hydration and activation before germination begins, ultrastructural modifications in the apertural region of pollen grains take place, in particular in intine layers[55]. Considering this initial inhibitory effect, it is remarkable that tube emergence of pollen exposed to our ELF-MFs during both rehydration and germination phases was significantly improved over stressed controls. Although it is known that alterations in $Ca^{2+}$ concentration are indisputably involved in modulation of pollen tube growth, the nature of these signals, how they are integrated, and the components upon which they act are still largely unknown and further experiments are required.

As regards the effects of indirect (water-mediated) treatment, the changes in physicochemical properties of water and water solutions under the action of static MF and EMF can be considered as a proven fact[25,47]; however, the question how such low-energy EMF radiation could modulate the functional activity of cells and organisms still remains unanswered. It is suggested that structural changes in EMF-induced water could serve as one of the possible primary targets through which the nonthermal biological effect of EMF is realized[56]. In our experiments, water present either in the rehydration humid chamber (relative humidity 100%) or in the growth medium can be involved; the molecules of water seem likely to mediate the interaction between ELF-MFs and the bio-object[56]. Moreover, we observed a repeated significant stimulatory effect on pollen performance only when magnetically induced water was subjected to the magnetic treatment for 24 h and used immediately after the exposure. It can be hypothesized that a wait of 1 h after exposure is still too long to maintain structural changes in water, at least in our experimental conditions. On the contrary, water irradiated with nonthermal MWs affected pollen germination not only when immediately used, but also several days after irradiation[50]. In fact, it has been reported[25] that the effects of magnetized water are related to the magnetizing time, to the intensity of the externally applied MF, and to the temperature of the water, even if without a linear relationship. The finding of an indirect action of ELF-MFs through water is the hardest to gain acceptance and it surely needs further experiments, carried out by different research groups, to prove the reproducibility of the effects.

Finally, since the main cell structures and functions are common to the majority of eukaryotes, we believe that our findings on pollen germination and growth could be of interest also from a medical point of view[35], at least as complementary to clinical studies, indicating that organisms, as coherent systems, are extraordinarily sensitive to weak intensity ELF-MF.

### ACKNOWLEDGMENTS

The authors are deeply grateful to Prof. Gian Lorenzo Calzoni (Department of Evolutionary Experimental Biology, Faculty of Science, Bologna University, Italy) for scientific care and fruitful discussion. The authors are also indebted to Dr. Antonio Gnes (ARPA, Regional Agency Environment Care, Ravenna, Italy) for assistance in statistical analysis.

The authors are also indebted to Dr. Sandro Pizzuti (ITEMA, Frascati, Roma, Italy) for assembling the EMF-generating device and to Dr. Giovanni Borghini for technical assistance.
